# “Conjugate Channeling” Effect in Dislocation Core Diffusion: Carbon Transport in Dislocated BCC Iron

**DOI:** 10.1371/journal.pone.0060586

**Published:** 2013-04-11

**Authors:** Akio Ishii, Ju Li, Shigenobu Ogata

**Affiliations:** 1 Akio Ishii Department of Mechanical Science and Bioengineering, Osaka University, Osaka, Japan; 2 Ju Li Department of Nuclear Science and Engineering and Department of Materials Science and Engineering, Massachusetts Institute of Technology, Cambridge, Massachusetts, United States of America; 3 Shigenobu Ogata Department of Mechanical Science and Bioengineering, Osaka University, Osaka, Japan; Jacobs University Bremen, Germany

## Abstract

Dislocation pipe diffusion seems to be a well-established phenomenon. Here we demonstrate an unexpected effect, that the migration of interstitials such as carbon in iron may be accelerated not in the dislocation line direction 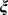, but in a conjugate diffusion direction. This accelerated random walk arises from a simple crystallographic channeling effect. 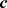 is a function of the Burgers vector ***b***, but not 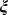, thus a dislocation loop possesses the same everywhere. Using molecular dynamics and accelerated dynamics simulations, we further show that such dislocation-core-coupled carbon diffusion in iron has temperature-dependent activation enthalpy like a fragile glass. The 71° mixed dislocation is the only case in which we see straightforward pipe diffusion that does not depend on dislocation mobility.

## Introduction

It is well appreciated that interstitial atoms and solutes can diffuse faster in extended defects such as dislocations or grain boundaries than in bulk lattice [1]–[10]. Because the dislocation looks like a pipe along the dislocation line direction 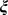, such fast transport is often called pipe diffusion [11]. The term implies the change in diffusivity tensor (rank-2) due to dislocation is locally enhanced in the longitudinal 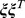 component. Here, 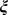 is three-dimensional column vector. Thus, 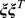 is rank-2 tensor. And the acceleration effect is comparatively small in the transverse directions:
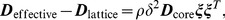
(1)where 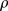 is the density of identical dislocations (assumed to be all running along 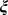), 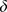 is some nominal diameter of the cylindrical pipes, and 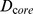 is a scalar. Such a mathematical model matches the symmetry of a cylindrical pipe; but real dislocations have vector charge ***b***, the Burgers vector. The theoretical problem also gets interesting when dislocations glide easily (low Peierls stress [12]) and can co-migrate with an interstitial atom. In this paper, we use atomistic simulations to address the detailed mechanism of dislocation-core-coupled carbon interstitial diffusion in iron. To our surprise, we found a large enhancement of carbon random walk in the 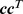 component:
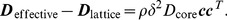
(2)where is three-dimensional column vector called the conjugate diffusion direction (CDD). Thus, there can be large enhancement in carbon transport transverse to 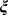. Even more strangely, c turns out to have nothing to do with 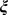, and is a function of the Burgers vector b and slip plane normal n only. Fundamentally, this core-enhanced interstitial diffusion arises from an atomic-geometry channeling effect that has some resemblance to ion channeling [13] phenomena.

## Results

**Figure 1:**
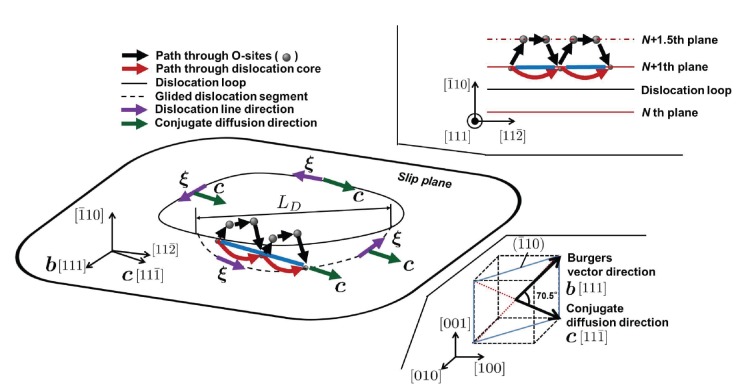
Illustration of conjugate channeling effect in dislocation core diffusion. Black arrows indicate the carbon diffusion path through bulk lattice (O-site) along 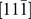 and black dots indicate the O-site along the path. Red arrows indicate the carbon diffusion path through dislocation core along 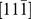. 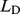 is the maximum dislocation core length which can be dragged by single carbon atom (See text for details). The dislocation loop is located between 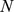
th and 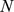
+1th plane as shown in upper right figure. Lower right figure show the relation between conjugate diffusion vector ***c*** and Burgers vector ***b***.

From the molecular dynamics (MD) and accelerated MD simulation trajectories, we observed surprising “transverse diffusion” of carbon at all temperatures, which means greatly accelerated random walk of the single interstitial along a direction that is not parallel to 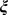. We denote this special random walk direction ***c***, the conjugate diffusion direction (please see FIG. 1: lower right); here 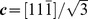, while 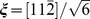, 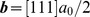. So instead of “longitudinal diffusion” or pipe diffusion along the dislocation core direction 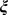, we locally observed a behavior described by Equation (2). This is made possible by the edge dislocation locally gliding to follow the carbon random walk everywhere (see Powerpoint S1). FIG. 1 illustrates the dislocation glide movement, following the trajectory of carbon diffusion (blue line) along 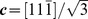 through the dislocation core (red arrow) and Octahedral (O) sites (black arrow). For O-site diffusion in a perfect lattice along ***c***, a carbon needs to move half plane higher than the 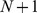
th atomic plane and pass through two O sites. On the other hand, carbon diffuses along arched path under the 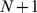
th plane when inside the dislocation core.

The co-movement of the edge dislocation core is due to extremely low Peierls barrier and lattice friction of edge dislocation in BCC iron (23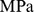 [4]). Because the binding energy of carbon with edge dislocation is 0.96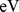 from our previous work [14], the carbon interstitial and a segment of the edge dislocation in fact form a co-diffusing “molecule” or a “complex” that undergoes random walk together under thermal fluctuations. The size of this “molecule” can be very roughly estimated as follows. One can plot potential energy’s lower envelop 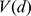 as one shifts the carbon atom from in the middle of the core (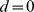) gradually to 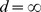. The maximum derivative
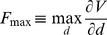
(3)is the Zener pinning force. Our calculations show that 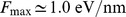 for the carbon-edge dislocation complex. With a Peierls stress 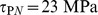, the Zener pinning force is able to drag along a dislocation core segment (FIG. 1) of length
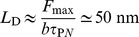
(4)with it. This means that not only dislocation can bias the movement of carbon as we know, but carbon can also affect the dislocation movement. Thus, we may need a paradigm shift in considering the relation between carbon and dislocation.

**Figure 2:**
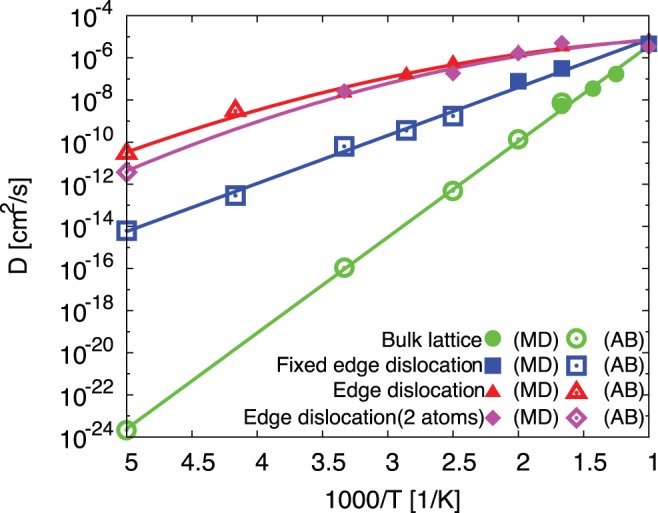
Temperature dependence of carbon diffusivity, 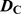 in 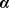-iron. MD and accelerated MD (AB) [14] results are displayed with filled and open symbols, respectively. One-carbon diffusivity results in bulk lattice, in fixed edge dislocation core, in free edge dislocation core are displayed with circles, squares, and triangles, respectively. Two-carbon diffusivity result in free dislocation are displayed with diamonds. The lines are polynomial fits as guide to the eye.

**Figure 3:**
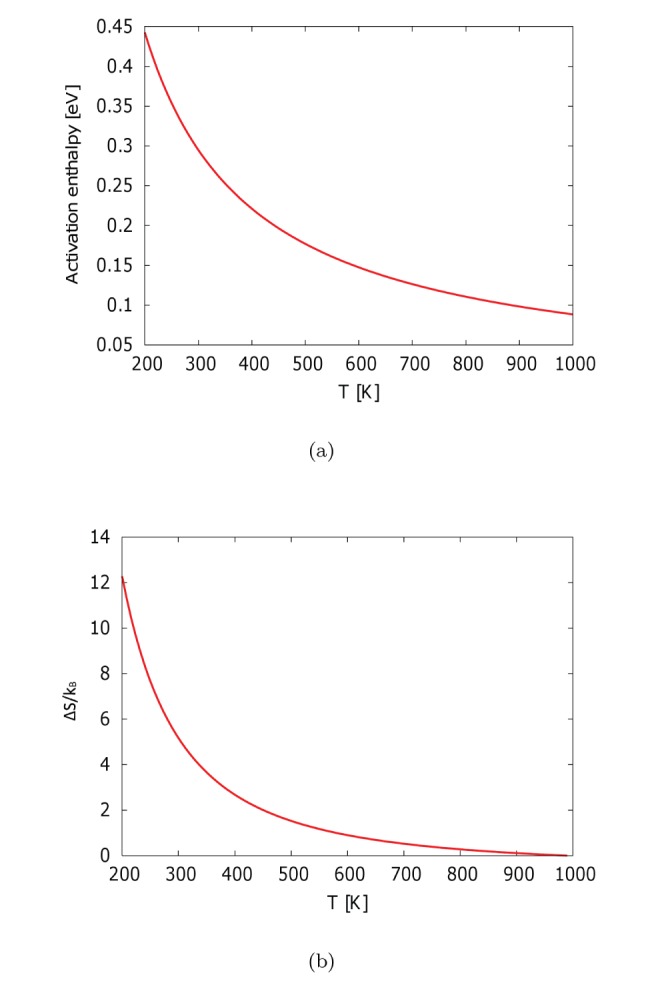
Temperature dependence of (a) activation enthalpy and (b) activation entropy of one-carbon diffusivity in free edge dislocation core.

From the MD and accelerated MD trajectories, we also calculated the temperature dependence of carbon diffusivity in the edge dislocation and compare with that in bulk lattice. 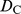 is defined as
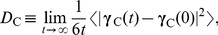
(5)

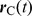
 is the carbon position at time 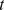. FIG. 2 shows the result of our calculation. 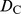 in the edge dislocation core (red line) is much higher than that in bulk lattice (green line). Further, the red line is not straight but has curvature against 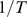. This means temperature-dependent activation free energy for dislocation-core-coupled carbon diffusion:
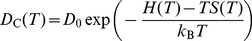
(6)where 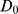 is a temperature-independent constant, 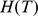 is the temperature-dependent activation enthalpy of diffusion, and 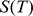 is the activation entropy. With the following Gibbs-Helmholtz equation at constant stress 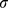,
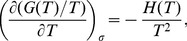
(7)the slope of FIG. 2 is
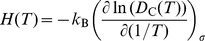
(8)which is plotted in FIG. 3(a). Then, by substituting 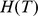 back into Equation (6), we can obtain 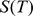.

In FIG. 3 (a), we see the activation enthalpy decreases rapidly in the low-temperature regime and becomes almost constant in the high-temperature regime. Correspondingly, as FIG. 3 (b) shows, 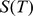 also decreases rapidly in the low-temperature regime and becomes almost constant at high temperatures. This behavior is quite similar to the temperature dependence of 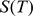 in the relaxation dynamics of fragile glass [15]–[17]. Here, we take 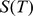 at 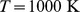 as reference entropy.

For contrast, the blue line in FIG. 2 shows 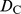 in the edge dislocation when we fixed the coordinates of all iron atoms along 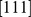 and 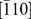. In this case, the blue line is linear like 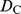 in the bulk lattice, so we do not see temperature-dependent activation enthalpy here. This means that the movement of dislocation is strongly related to the “fragility”. The temperature variation of diffusive activation parameters 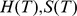 could arise from something akin to a gradual phase transition, where carbon diffusion in the dislocation at high temperature is not like the diffusion in the solid but more like diffusion in the liquid.

We also performed MD simulation with two carbon atoms in the same edge dislocation core model. The diffusion of each carbon atom along 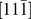 becomes slower than the case of one carbon in the same model, particularly at low temperature (FIG. 2). This is because the two carbon atoms and dislocation interact by long-ranged elastic interactions and start to take on a many-body character, in the way of “carbon-dislocation core-carbon” complexes. At higher temperature, the carbons and dislocation move independently. Clear “complex” feature cannot be found any more.

**Figure 4:**
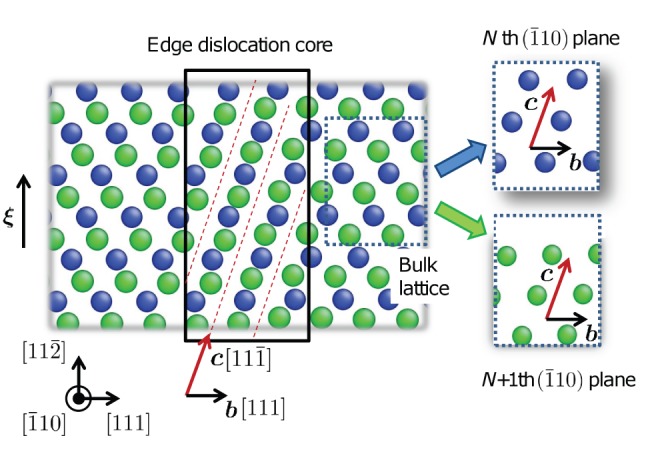
Conjugate diffusion avenue in edge dislocation core. Blue and green dots indicate the atoms on 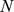
th and 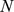
+1th 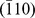 planes, respectively. Red dashed lines indicate the conjugate diffusion avenue in the dislocation core, a channel as wide as possible (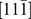 is one of the two widest avenues on a plane) and as tall as possible (a full 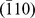 interplanar spacing) in a BCC crystal.

We performed geometrical analysis to explain “transverse core diffusion” in BCC metals. We took two adjacent 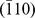 planes, the 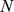
th and 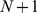
th, between which the edge dislocation core (FIG. 4: left) sits. As shown on FIG. 4: right, each 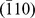 plane contains two equally wide ‘avenues’, that runs along 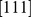 and 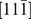, respectively. (There is also a narrower avenue that runs along 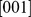, which we ignore here). Now pay attention to the 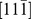 avenue. Without the dislocation, the 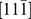 avenue on plane 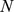 and the 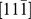 avenue on plane 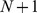 are collinear, but staggered, thus hindering each other. A carbon interstitial would live midway between plane 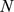 and 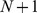, and because of the staggering would be trapped in deep wells, which are the Octahedral (O) sites of a perfect lattice. Climbing out of one of these O-site is energetically costly, before it can move into another O-site, and thus carbon diffusion in a perfect lattice is sluggish. The same is true for the 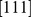 avenue on each plane. However, when an edge dislocation is present, plane 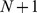 would be shifted along relative to plane 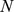, and ***b*** happens to be 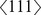. This shift does not help the 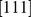 avenues, which remain staggered between the two planes even in the dislocation core, since translating an avenue along its own running direction cannot unstagger two avenues on two planes. But, such a shift can open up the *conjugate* avenue, 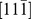, since ***b*** is *not* parallel to this, equally wide, avenue. In particular, when the shift is exactly 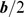, as at the center of the edge dislocation core, the 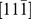 avenue on plane 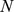 and the 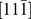 avenue on plane 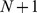 are completely unstaggered, as shown in FIG. 4: left. This creates a channel of percolating free volume along 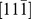 that is as wide as possible (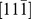 is one of the two *widest* avenues on a plane), and as tall as possible (a full 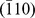 interplanar spacing). Thus, it should be no surprise that interstitial carbon, with its own excess volume, would find random walk along the 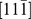 channel in the core particularly easy. The low Peierls barrier and easy glide of edge dislocation, as reflected by Equation (4), further provide a “rolling carpet” for carbon.

We have also computed the 0 K activation energy barrier along the fast diffusion channel using first-principles density functional theory (DFT) calculation, and obtained an energy barrier of 0.14 eV along CDD, which is much lower than the 0.86 eV barrier [18] along O-site diffusion path in bulk lattice. For reference, the EAM potential used here gave activation energy barrier of 0.39 eV along CDD. Thus, our geometric analysis stands even if detailed chemical bonding and magnetism are considered.

## Discussion

Based on the analysis above, we can make several comments. First, the degeneracy of such conjugate diffusion direction(s) is 1, at least for 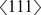 slip dislocations on BCC 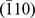, which admits two Burgers vectors on the same plane, 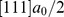 and 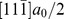. If 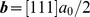 slip is activated on the plane, then the 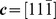 channel opens up. If the 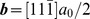 slip is activated on the plane, then the 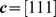 channel opens up - thus the term ‘conjugate’. For geometric reason outlined above, ***c*** can never be ***b*** itself. Lothe mentioned that facile core diffusion requires at least “two strings of easy diffusion” or “two high-diffusivity paths” in the dislocation core [11], [19]. A degeneracy of 1 for the dominating CDD has important macroscale consequences on carbon transport, as will be outlined below.

It might also be possible to apply the same geometric analysis to some other crystallographic avenues (narrower) on BCC 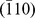 plane, some other planes of BCC which have different avenues, or some other crystal lattice like FCC or HCP, where the degeneracy of conjugate diffusion directions 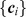 may exceed 1 for the same 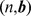. But in this first paper, we do not want to elaborate on those scenarios, since the carbon transport behavior in dislocated BCC iron seems to be dominated by a single conjugate channel, the so-called “string of easy diffusion” or “high-diffusivity path” [11], [19]. We want to discuss the consequence of this more fully, before moving onto higher-order effects.

**Figure 5:**
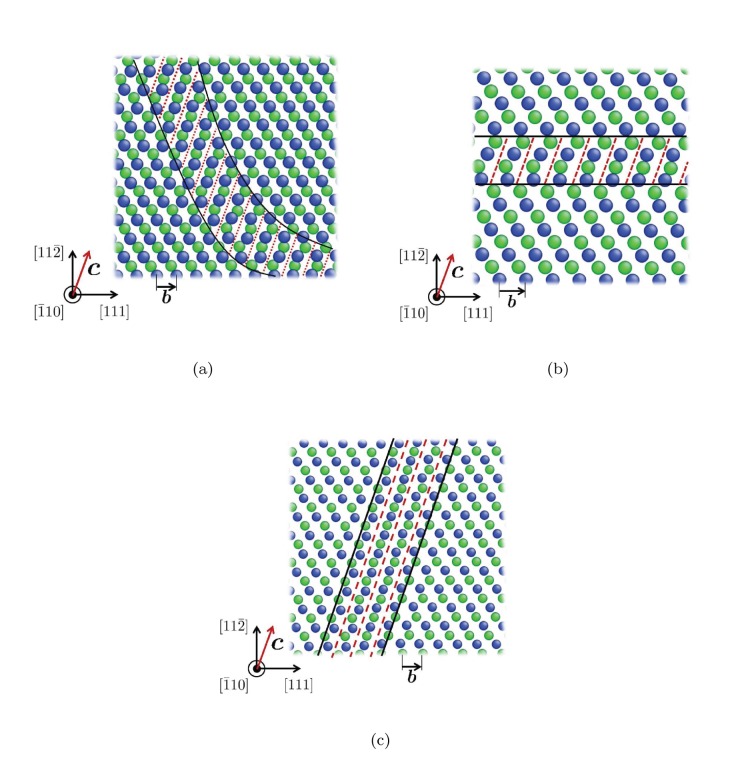
Geometries of (a) curved dislocation core, (b) screw dislocation core, and (c) 71° mixed dislocation core along 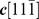. Blue and green dots indicate the atoms on 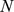
th and 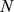
+1th 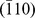 planes, respectively. Black lines are the eye guides for the dislocation cores. Red dashed lines indicate the conjugate diffusion avenue along 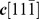 in the dislocation core, a channel as wide as possible (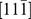 is one of the two widest avenues on a plane) and as tall as possible (a full 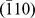 interplanar spacing) in a BCC crystal.

Second, the dislocation line direction 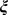 does not feature prominently in the analysis yet. Indeed, the geometric argument we put forth above regarding “staggering”, “unstaggering” and “channeling” could be seen at the level of the generalized stacking fault (GSF) [20] calculation, for which a large supercell calculation is not needed. The consequence of this observation is a peculiar effect, shown in FIG. 1, that all segments of a glide dislocation loop share the same conjugate diffusion direction (the green arrows). This has been verified by direct MD and accelerated MD simulations for mixed dislocations and screw dislocation on 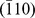 plane. We used a BCC crystal of 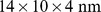 (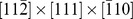) with free surfaces for every boundary. We made one curved, mixed dislocation and one screw dislocation on the 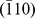 plane in this model and took the 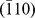 plane which contains the dislocation core and its neighboring plane, same as the case of the pure edge dislocation. As shown in FIG. 5 (a) and (b), the two unstaggered avenues and channel along 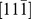 clearly appears in the mixed dislocation and screw dislocation cores also. We have also computed 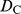 in the mixed dislocation at 300 K to be 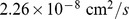, which is nearly identical to 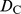 in the edge dislocation core at 300 K, 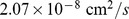 (FIG. 2). Thus, transverse core diffusion along CDD is a general phenomenon for a single interstitial atom, irrespective of 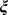. For the screw dislocation, however, even though we still see the interstitial atoms jittering back and forth along, the mobility of screw dislocation is so low that there is no rolling-carpet effect.

Based on the geometrical reasoning above, the “mean core path” of a single carbon interstitial inside a general, mixed dislocation core can be approximated as
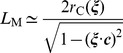
(9)where 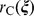 is the core radius of the dislocation with line direction 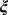, and the denominator is the directional sine between 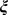 and the conjugate diffusion direction that does not depend on 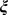. 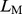 is how much a single interstitial can move on its own along the core channel, without requiring dislocation mobility to kick in (“rolling carpet”) for the next step.

Third, a special case arises when Equation (9) encounters a singularity, that is, when 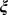 is parallel to ***c*** and 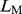 diverges. In this special case and special case only, dislocation mobility is not required, “transverse core diffusion” disappears, and totally longitudinal core diffusion (pipe diffusion) is easily observed. For BCC metals, these special dislocations are 71° mixed dislocations. These 71°-dislocations form “super highways” for interstitial diffusion. Even if these dislocations were immobile, they can still greatly accelerate mass transport (FIG. 5(c)).

Fourth, for non-71°-dislocations, high dislocation mobility is a pre-requisite for accelerated transverse core diffusion. Added onto this dislocation mobility requirement is an intriguing elastic-energy consideration. The motion of any glide dislocation creates inelastic strain, or transformation strain, 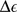, localized on the areas swept. If the glide dislocation can follow the random motion of interstitials, random patches of inelastic strain would also be created. In our MD and accelerated MD calculations, we have not applied any external stress thus far. So the work term 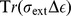 is zero, and the random inelastic strain due to carbon random walk is not energetically biased. However, suppose 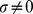, the coupling between dislocation core and carbon movement would mean the random walk of carbon is no longer unbiased, namely the probability of forward jump 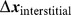 would no longer be equal to the probability of backward jump 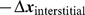, and the drift-diffusion of carbon would start to take on a vector-charge character. This is expected, as the co-diffusing “molecule” consists of a scalar-mass-charged interstitial coupled to vector-charged dislocation. If there are equal numbers of oppositely signed dislocations, then this vector-charged character of carbon transport may cancel out macroscopically. But, if there are geometrically necessary dislocations (density of plus signed dislocations is statistically different from that of opposite signed dislocations), then mass transport of carbon could be severely influenced by the GND density.

Fifth, extending the above elastic energy considerations, when there is more than one carbon interstial in the dislocation core, the different patches of 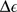 field would start to interact by long-ranged elastic interactions. This means carbon diffusion may no longer be mainly a single-carbon decision, but start to take on a many-body character, in the way of “carbon-dislocation core-carbon” complexes. However, fast carbon diffusion are still observed in the special 71°-dislocations no matter how many carbon atoms we put in, because dislocation core motion is not required in these dislocations. We have computed 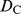 in 71°- dislocation using the simulation cell of mixed dislocation, which is 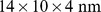 (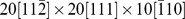) with fixed surface for every boundary. We insert one or two carbons as interstitial atom in the dislocation core. 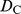 of two carbon model at 200 K to be 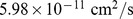, which is nearly identical to 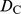 of the one carbon model at 200 K, 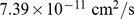. Thus, number density of carbon atom along dislocation core does not have great influence over 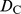 in 71°-dislocation.

In summary, based on atomistic simulations and crystallographic analysis, we have observed true longitudinal “pipe diffusion” only in the case of 71°-dislocation in BCC iron, when the dislocation line is aligned with the conjugate diffusion direction. In all other cases when the dislocation line is misaligned with the conjugate diffusion direction, we have observed core-coupled “transverse core diffusion” with rolling-carpet like motion, if the dislocation mobility is high enough, where atomic motions of diffusive and displacive characters are closely coupled. [21] This is a big surprise since our results seem to not say anything definitive about the concept of “pipe diffusion” for non-71°- dislocations. We cannot say that pipe diffusion is absent for these dislocations. Our simulation results just seem to suggest that at the fundamental atomic level, the “transverse core diffusion” is the first-order effect, and “pipe-like diffusion” of interstitial carbon, if it exists, may be a many-body and higher-order effect in these non-71°-dislocations.

## Methods

For molecular dynamics calculation, an edge dislocation with 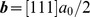, 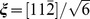 was created in BCC iron at the center of an atomistic simulation cell, which is 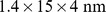 (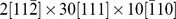). This dislocation lies on the 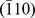 plane (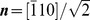) of BCC iron, as all dislocations in this paper. This model has free-surface boundary conditions along 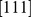 and 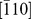, and periodic boundary condition along 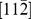. We then insert one or two carbons as interstitial atom in the dislocation core, and apply both molecular dynamics (MD) and accelerated molecular dynamics [14] simulation to study dislocation-core-coupled carbon transport. For the simulations, we used our own MD code and Nosé-Hoover thermostat [22], [23]. An embedded-atom model (EAM) potential of the Fe-C system [24] was used. Before MD simulation, we performed structural relaxations [25] so we have well-defined starting configurations. MD was used for temperature 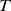 ranging from 200 K to 1000 K.

For lower temperatures (200 K, 300 K), we used accelerated MD (Adaptive Boost [14]) method. In this method we add a boost potential 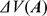 to the Hamiltonian in order to reduce the activation-energy barriers for rare events. Their occurrences are accelerated by a common factor, which can be estimated based on the transition state theory (TST) [26]. ***A*** is collective variables defined as 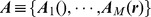 where ***r*** is atomic position.


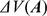
 is defined as a function of the collective variables:
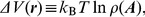
(10)where 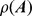 is probability density. In a canonical ensemble (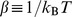) described by the Hamiltonian 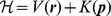, where 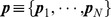 is momenta, the probability density 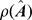 of those collective variables is



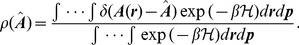
(11)In this study, MD simulation in the canonical ensemble is performed for 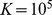 time steps to obtain the 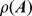. We used density estimator [27] to estimate smooth histogram 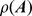 from the finite MD sampling. Projection of coordinate of carbon atom and coordinate of two-carbon mass center to the dislocation line 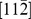, 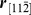 are taken as the collective variable for the one carbon and two carbon diffusion simulations in the edge dislocation core. simultaneously.

For DFT calculation, the geometry of diffusion channel in 71° mixed dislocation core (see FIG. 4) was modeled by a supercell, which is 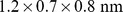 (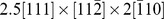) with 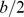 relative shift of adjacent 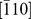 planes in Burgers vector direction 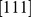. We put one carbon atoms between the shifted adjacent planes and relaxed the atomic structure subject to the constraint of 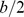 shift. Then the carbon atom was moved along the CDD with relaxing atomic structure subject to the constraint of carbon atom coordinate along CDD and center of mass of the model. We used the Vienna Ab-Initio Simulation Package [28]. Perdew-Wang generalized gradient approximation (GGA) [29], projector augmented-wave (PAW) method [30] and 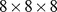 Monkhorst-Pack Brillouin zone (BZ) k-point sampling [31] were used.

## Supporting Information

Powerpoint S1 **Movie of edge dislocation glide concurrent with carbon random walk in BCC Fe (200 K, accelerated MD).**
(PPTX)
