# Supplementary material for: “Conjugate Channeling” Effect in Dislocation Core Diffusion: Carbon Transport in Dislocated BCC Iron
Source: PLoS One. 2013 Apr 11;8(4):e60586. doi: 10.1371/journal.pone.0060586 (PMC3623912; doi:10.1371/journal.pone.0060586)
Supplement: Powerpoint S1 — Movie of edge dislocation glide concurrent with carbon random walk in BCC Fe (200 K, accelerated MD). (PPTX) [file pone.0060586.s001.pptx]

## Slide 1
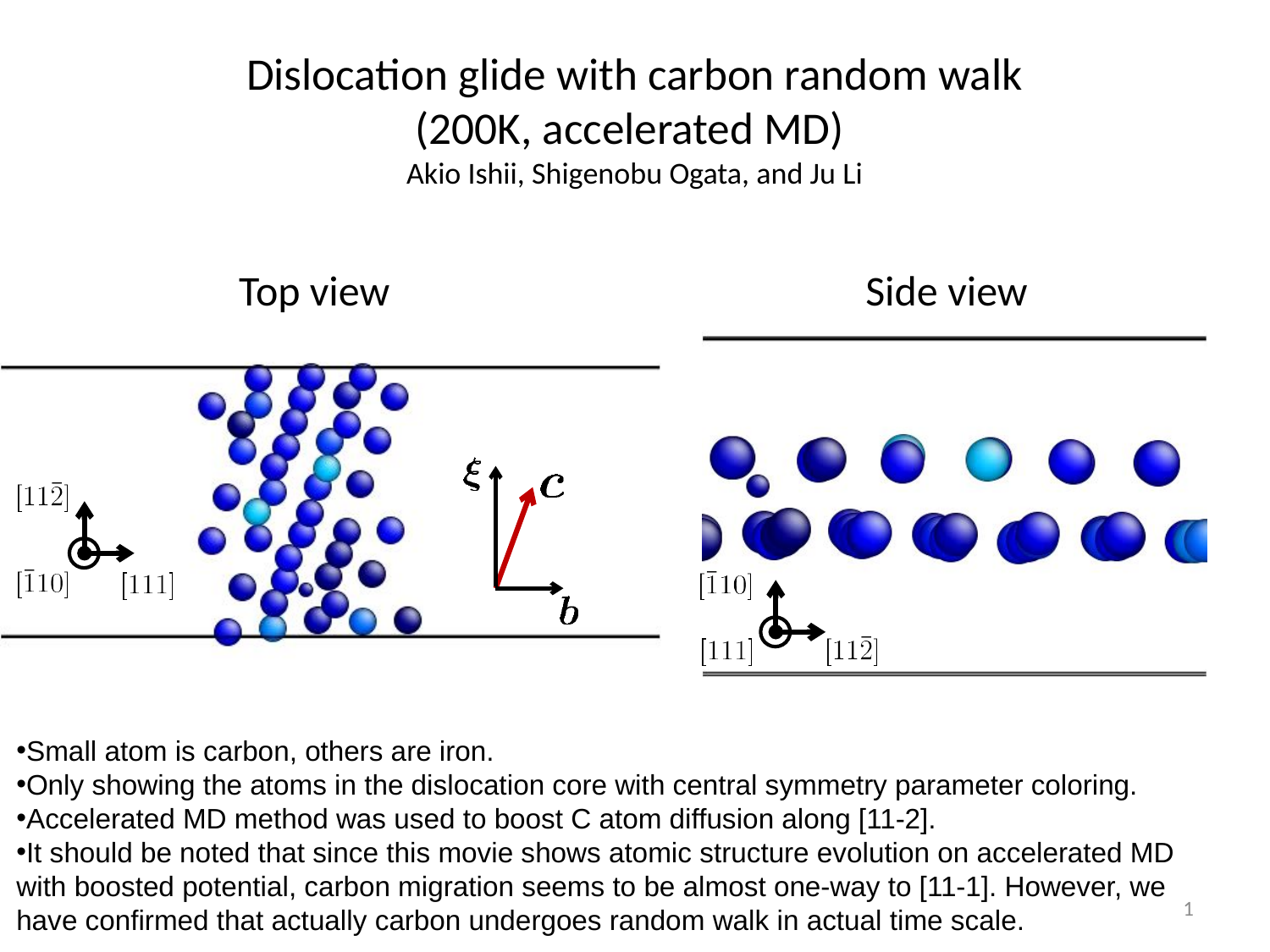

# Dislocation glide with carbon random walk (200K, accelerated MD) Akio Ishii, Shigenobu Ogata, and Ju Li
Side view
Top view
Small atom is carbon, others are iron.
Only showing the atoms in the dislocation core with central symmetry parameter coloring.
Accelerated MD method was used to boost C atom diffusion along [11-2].
It should be noted that since this movie shows atomic structure evolution on accelerated MD with boosted potential, carbon migration seems to be almost one-way to [11-1]. However, we have confirmed that actually carbon undergoes random walk in actual time scale.
1
